# Antibacterial micro/nanomotors: current research progress, challenges, and opportunities

**DOI:** 10.7150/thno.92449

**Published:** 2024-01-01

**Authors:** Xin-Yang Liu, Rui-Fang Li, Jun Jia, Zi-Li Yu

**Affiliations:** 1 State Key Laboratory of Oral & Maxillofacial Reconstruction and Regeneration, Key Laboratory of Oral Biomedicine Ministry of Education, Hubei Key Laboratory of Stomatology, School & Hospital of Stomatology, Wuhan University.; 2 Department of Oral and Maxillofacial Surgery, School and Hospital of Stomatology, Wuhan University, 430079 Wuhan, China.

**Keywords:** micro/nanomotors, bacterial infections, challenges, opportunities

## Abstract

Bacterial infections remain a formidable threat to human health, a situation exacerbated by the escalating problem of antibiotic resistance. While alternative antibacterial strategies such as oxidants, heat treatments, and metal nanoparticles (NPs) have shown potential, they come with significant drawbacks, ranging from non-specificity to potential environmental concerns. In the face of these challenges, the rapid evolution of micro/nanomotors (MNMs) stands out as a revolutionary development in the antimicrobial arena. MNMs harness various forms of energy and convert it into a substantial driving force, offering bright prospects for combating microbial threats. MNMs' mobility allows for swift and targeted interaction with bacteria, which not only improves the carrying potential of therapeutic agents but also narrows the required activation range for non-drug antimicrobial interventions like photothermal and photodynamic therapies, substantially improving their bacterial clearance rates. In this review, we summarized the diverse propulsion mechanisms of MNMs employed in antimicrobial applications and articulated their multiple functions, which include direct bactericidal action, capture and removal of microorganisms, detoxification processes, and the innovative detection of bacteria and associated toxins. Despite MNMs' potential to revolutionize antibacterial research, the translation from laboratory to clinical use remains challenging. Based on the current research status, we summarized the potential challenges and possible solutions and also prospected several key directions for future studies of MNMs for antimicrobial purposes. Collectively, by highlighting the important knowns and unknowns of antimicrobial MNMs, our present review would help to light the way forward for the field of antimicrobial MNMs and prevent unnecessary blindness and detours.

## 1. Introduction

Bacterial infections persist as a critical global health concern, accounting for a significant proportion of morbidity and mortality 1. In 2019, they were recognized as the second leading cause of death worldwide, surpassed only by ischemic heart disease and comprising one-eighth of all deaths globally 2. Traditional antimicrobial strategies, chiefly antibiotic therapy, necessitate frequent and high dosages, often producing an array of side effects 3. Moreover, widespread and sometimes indiscriminate antibiotic use has led to the emergence and dissemination of antibiotic-resistant strains, thereby narrowing the range of effective therapies and posing a grave threat to public health 4. Although alternative antibacterial therapies, including metal-based nanomedicines, oxidants, heat, antimicrobial peptides, and catalytic treatments, have been explored, overcoming infections effectively remains daunting. Such alternatives can lack specificity and inadvertently damage both human tissues and the environment. Presently, conventional methods for bacterial detection, like culture-based techniques and polymerase chain reaction (PCR), demand complex equipment, specialized skills, and extended processing times 5. This has prompted researchers to explore more refined and faster antimicrobial approaches alongside developing simpler, more sensitive diagnostic assays.

Recent strides in nanotechnology have ushered in micro/nanomotors (MNMs) with self-propulsion abilities. Engineered to convert various forms of energy into propulsive force, MNMs initially relied on inorganic catalysts acting on chemical fuels, raising biocompatibility and toxicity concerns. This sparked the development of enzyme-based and fuel-free MNMs reliant on external stimuli such as sound, light, and electricity. More recently, a focus on inherently motile cells and bacteria, such as sperm, microalgae, and flagellated bacteria, has led to the burgeoning field of biohybrid MNMs. Transitioning energy sources has broadened MNMs' applicability in biomedicine, especially in the field of antibacterial. Their remarkable self-propulsion allows for quick and efficient bacterial binding and biofilm penetration. By carrying drugs or nanoparticles, MNMs deliver their antimicrobial effects with minimal collateral damage. Furthermore, receptor-functionalized MNMs exhibit enhanced capacities for rapid bacterial capture and real-time monitoring. MNMs are notably effective not only in treating bacterial inflammations but also in combating bacteria-induced cancer or other diseases. For example, infections by Helicobacter pylori, a leading risk factor for gastric malignancy, are also an area where MNMs show promise 6, 7. Capable of converting acidic environments into driving force, MNMs locally adjust pH levels, triggering drug release, and thus potentially restoring gastric balance and diminishing H. pylori populations 8. This innovative modality offers a novel therapeutic avenue for gastric cancer management.

**Figure 1:**
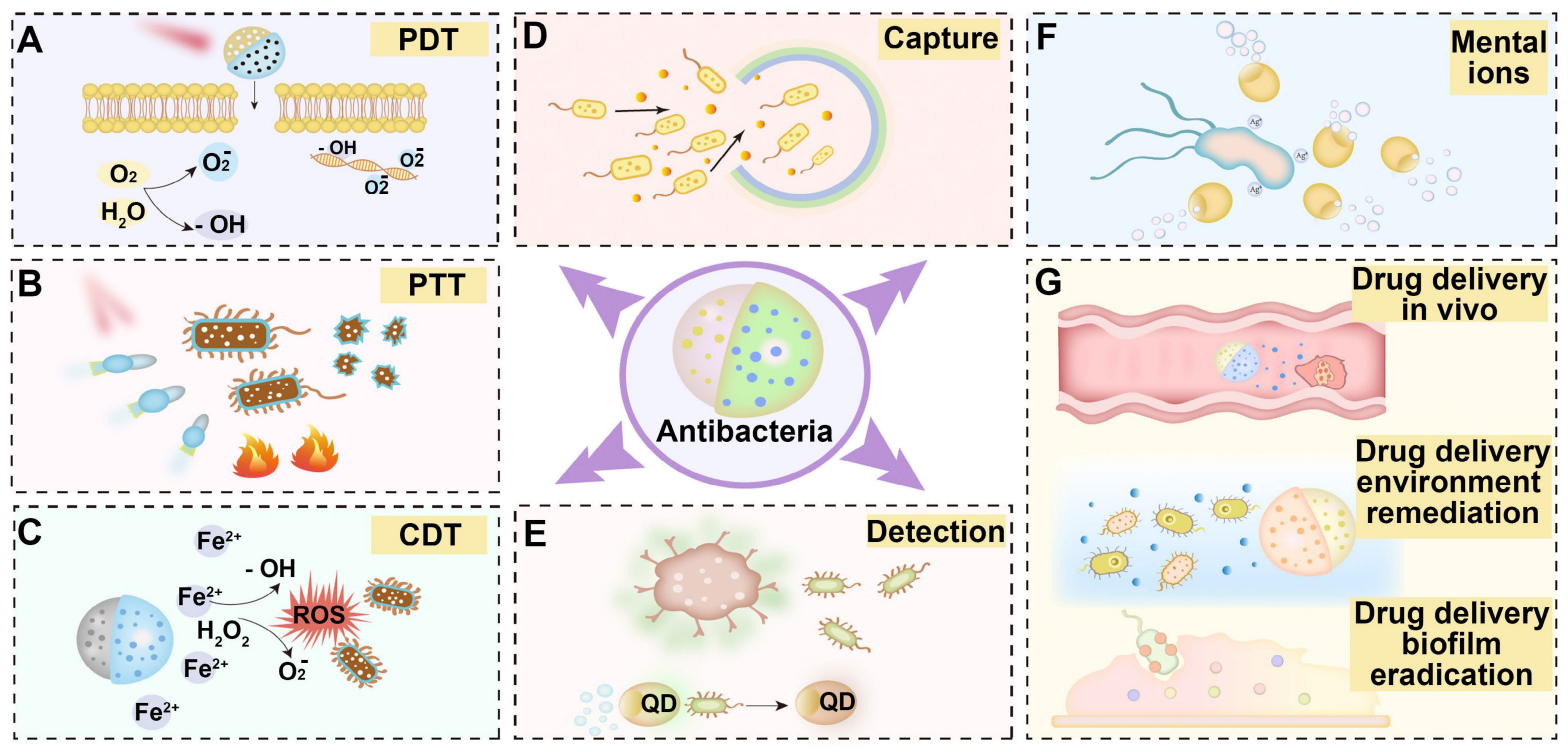
Schematic diagram of micro/nanomotors based on different antibacterial mechanisms. **(A)** MNMs for photodynamic therapy. **(B)** MNMs for photothermal therapy. **(C)** MNMs for chemodynamic therapy. **(D)** MNMs for capture microorganisms. **(E)** MNMs for bacterial detection. **(F)** MNMs for bactericidal with mental ions. **(G)** MNMs for drug delivery in vivo, environment remediation and biofilm eradication.

In this review, we provide a comprehensive overview of the propulsion system of antibacterial MNMs and their critical roles in antibacterial activities, including drug delivery, photothermal therapy, photodynamic therapy, chemodynamic therapy, as well as sterilization through metal ions (**Figure 1**). We also delve into their utility in microbial capture and detoxification and evaluate them as innovative platforms for bacterial detection. While MNMs have significantly advanced antibacterial research, their transition from laboratory to clinic faces persistent hurdles. This prompts us to discuss the prospective challenges and outlooks that merit attention: Controlled drug release at specific sites within the body using MNMs; Combined effects of multiple bactericidal mechanisms based on MNMs; Improve the accuracy, sensitivity and convenience of MNMs detection methods; The improvement of antibacterial MNMs propulsion system and structural design.

## 2. Propulsion system of antibacterial micro/nanomotors

### 2.1 External field-driven micro/nanomotors

#### 2.1.1 Light

Light serves as a pivotal energy source that is both sustainable and easily regulated. The propulsion of light-driven MNMs typically hinges on the integration of light-responsive molecules and nanomaterials, which transduce photonic energy into mechanical force.

**Near-infrared (NIR) light**. The principal propulsion mode of NIR light-driven MNMs is self-thermophoresis, a process where light-absorbing materials generate heat upon NIR exposure, producing a local temperature gradient that propels the MNMs. This thermophoretic propulsion necessitates the design of Janus particles with asymmetrical surfaces offering differential light absorption capabilities. When illuminated, the resultant temperature differential across the particle leads to the local thermal movement of fluids, driving the MNMs in the direction of the temperature gradient 9. Alexandre et al. have developed a light-driven Janus motor composed of mesoporous silica NPs with a gold hemisphere and half-capped with cysteamine 10. When exposed to NIR light, the gold nanolayer becomes thermally active, allowing the Janus motor to move at ultrafast speeds and exhibiting rapid and efficient photothermal ablation capability against Escherichia coli (E. coli). Additionally, such NIR-light-driven MNMs excel not only at neutralizing free-floating bacteria but also at dismantling biofilms (notoriously resistant bacterial communities). Tijana's group innovated a nanomotor that, under NIR illumination, exhibits robust self-propulsion due to the gold constituent's photothermal effect 11. This enables the motor to penetrate and disrupt biofilm structures, significantly aiding in their eradication even with minimal light exposure (30s-3min). Moreover, NIR-light-driven dual plasmonic nanomotors (AuNR-SiO_2_-Cu_7_S_4_) have been crafted for enhanced antibacterial action 12. These nanomotors profit from the vigorous plasmonic interaction between gold nanorods and Cu_7_S_4_, amplifying energy transfer processes. They can move at a high velocity (~9.8 μm/s) under a low-power NIR-II laser, which promotes deeper transdermal reach and efficient targeting of bacterial cells.

**Visible-light (VIS)**. In visible-light-powered MNMs, the propulsion mechanism often involves self-electrophoresis or self-diffusiophoresis. Self-electrophoresis relies on creating a local electric field and an asymmetric ionic distribution to mobilize the particles. Conversely, self-diffusiophoretic MNMs leverage solute concentration gradients across their surface to induce fluid movement, thereby propelling themselves 13. Katherine Villa and colleagues have constructed single-component BiVO_4_ micromotors that operate under visible light. These micromotors can swim solo or cluster collectively, adeptly attaching themselves to yeast cell membranes 14. Further, Marta et al. have reported a micromotor using the biocompatible polymer polycaprolactone (PCL) to encapsulate CdTe or CdSe@ZnS quantum dots (QDs) as photoactive supplies 15. These micromotors spring into action under VIS light (470 to 490 nm), which is propelled through a diffusiophoretic mechanism. They maneuver rapidly through challenging environments such as human blood and notably enhance the efficiency and speed of toxin removal compared to non-quantum dot-equipped motors 16. Imbued with exceptional biocompatibility and increased motility, light-driven nanomotors demonstrate formidable antibacterial effectiveness. Their adaptability makes them ideal candidates for various applications, encompassing detoxification, drug delivery, microbial capture, biosensing, and wastewater treatment, particularly beneficial for purifying microbial-contaminated waters. Thus, light-driven MNMs hold immense promise for ushering in novel solutions across diverse domains.

#### 2.1.2 Magnetic field

Electric currents interacting with magnetic materials can generate a magnetic field, a principle that lays the foundation for fuel-free propulsion at the nanoscale. By coating micro/nanostructures with ferromagnetic metals like nickel or iron, magnetic MNMs gain the capacity for movement and can be precisely guided using an externally applied magnetic field. The control over these MNMs is refined by adjusting the magnetic field's intensity and frequency, ultimately altering their speed and trajectory 17. The propulsion mechanism of flexible magnetic micro/nanoswimmers relies on the asymmetrical deformation of elastic filaments 18. The swimmers' motion is engendered by a non-uniform magnetic field, while helical magnetic MNMs are propelled through a rotational motion induced by a dynamic magnetic field that mimics the corkscrew motion, propelling these structures efficiently through fluids 19. Safeguarded by their biocompatibility, such MNMs can interact within biological environments without causing damage, as the weak magnetic fields required for their operation are non-invasive to cellular structures and tissues 20.

**Propulsion**. Innovative strides in this arena include the development of a helical nanomotor enveloped with a natural platelet membrane, inventively operated by magnetic fields 21. The application of a platelet-membrane coating notably enhances the nanomotor's affinity for binding bacterial toxins and pathogens. With magnetic guidance, these nanorobots can attach to and remove bacteria, paving the way for potential use in biodetoxification and targeted treatment against infectious diseases, merging biological functionality with advanced locomotion. Moreover, magnetically actuated plant-based micromotors were also have been innovated, exemplified by Bhuyan et al.'s creation of T-Budbots derived from tea buds 22. These biocompatible micromotors navigate biofilm matrices under magnetic guidance, enabling precise biofilm disruption and removal, a pioneering “Kill-n-Clean” strategy that shows significant promise for therapeutic and environmental biofilm eradication.

**Guidance**. Magnetic fields not only power propulsion but also act as superb means for the steerable guidance of MNMs. Once equipped with propulsion systems like enzymatic drives or catalysts for chemical fuel, ferromagnetic nanoparticles (NPs) incorporated into MNMs can be directed along predefined paths influenced by an external magnetic field. For example, magnetically-guided ultrasound-powered nanowire motors can follow intricate routes and carry substantial loads 23. Magnetic guidance also enhances the capabilities of self-propelled Janus microbots engineered to purify contaminated water by collecting bacteria 24. After successfully navigating water and entrapping bacteria, a magnetic field can retrieve these microbots, facilitating the removal of the captured bacteria and ensuring water decontamination.

#### 2.1.3 Ultrasound

For a considerable time, scientists have been aware of the ability of ultrasound to induce movement in MNMs 25. Ultrasound is sound waves with a frequency higher than the upper audible limit of human hearing. The most common method of ultrasonic manipulation of micro/nanoparticles is based on the ultrasonic propulsion of metal nanorods with shape asymmetry 26. When ultrasound waves are emitted, they can interact with any objects or particles they encounter. In 2012, Mallouk and colleagues demonstrated the use of ultrasound as a propelling force for MNMs, attaining speeds up to 200 μm/s 27. Ultrasound-propelled MNMs are widely used in antibacterial applications due to their excellent biocompatibility, precise controllability, and ability to operate without chemical fuel or external magnetic fields. Kiristi et al. have developed an innovative approach by integrating lysozyme with ultrasound-driven porous gold nanowire motors to target bacteria 28. The noticeable enhancement in antibacterial effectiveness can be attributed to the constant motion of the nanomotors under ultrasonic activation. This motion promotes efficient fluid mixing, significantly increasing the interaction between lysozyme and bacteria and reducing the risk of passivation effects. Such fuel-free, ultrasound-propelled nanofighters demonstrate a pronounced increase in antibacterial proficiency and expeditious bacterial neutralization compared to stationary systems. Garcia-Gradilla et al. also have constructed magnetically-guided three-segment Au-Ni-Au nanowire motors, which are pushed through the use of acoustic forces generated by a piezoelectric transducer 23. Their research showcases that these acoustically-driven nanomotors exhibit enhanced capabilities, performance, and functionalities. The potential applications of these versatile devices include magnetic navigation, coordinated movement, cargo transport, biological separation, drug delivery, and much more.

This acoustically-driven nanomotor, which possesses excellent biocompatibility and motion capabilities, opens new possibilities in the field of antibacterial applications. With its unique functionality and controllability, these devices can achieve targeted drug delivery and treatment at the microscopic scale, facilitating precise treatment of infection sites. Furthermore, the acoustic waves can be used to manipulate the motion trajectory and speed of the nanomotors within the biological body, thus enhancing effectiveness of treatment. Further exploration and development are necessary to resolve antibacterial challenges more effectively.

### 2.2 Enzyme-driven

Enzymes play a crucial role as biological catalysts, exhibiting a remarkable capacity to transform chemical energy into mechanical power inside living organisms. Utilizing enzymes as biocatalysts in order to supply the propelling force is an innovative approach. The fuel of enzymatic reaction is biofriendly, rendering enzyme-powered micro/nanomotors (EMNMs) particularly advantageous in the realm of biomedicine owing to their intrinsic biocompatibility 29. EMNMs show a broad spectrum of antimicrobial applications, such as targeted drug delivery 30, 31, biofilm eradication 32, bacterial capture and biosensing 33.

**Catalase**. Catalase stands out as an early model for EMNMs. Its capacity to decompose hydrogen peroxide (H_2_O_2_) into water and oxygen gas yields a forceful propelling action, resulting in impressive propulsion of these MNMs. Zhao et al. have constructed a Janus micromotors for motion-capture-lighting of bacteria 33. Catalase is grafted onto one side of the Janus fiber rods to facilitate the generation of oxygen bubbles, which serve as the propellant for micromotors. Conversely, mannose is conjugated to facilitate precise identification of FimH proteins located on the outer layer of bacteria. Compared to static-counterpart, catalase-driven nanomotors can enhance the sensitivity, selectivity, and reliability of monitoring E. coli. Particularly, when the aspect ratio of the motor is 2, it exhibits significantly higher mean square displacement while maintaining directed motion trajectories, resulting in improved bacterial capture and greater fluorescence intensity changes. While catalase-driven motors can achieve rapid movement by generating oxygen gas bubbles for propulsion, it is important to note that H_2_O_2_ exhibits toxicity towards living cells and can have detrimental effects on organisms.

**Urease**. Due to the due to the potential cytotoxicity of H_2_O_2_, there has been a pivot toward the use of urease, a more biocompatible catalyst. Urease enzymatically liberates ammonia and carbon dioxide from urea, creating a gradient that powers the nanomotors via a self-propulsion phoretic mechanism. This safer propulsion means urease-powered MNMs are making waves in research for bacterial eradication within organisms. Tang's group presented a study on a Janus platelet (JPL) micromotor that utilizes an endogenous enzyme to power its movement, involving the immobilization of urease onto the surface of natural platelet cells 31. The utilization of urea fuel significantly boosts the propulsion performance of JPL-motors, resulting in enhanced biological targets binding efficiency. Consequently, the therapeutic effectiveness of JPL-motors is improved when they are utilized to deliver anticancer or antibacterial medicines. In addition, Vilela and co-workers have developed nanomotors composed of urease-functionalized mesoporous silica nanoparticles (U-MSNPs), which are used to eliminate non-pathogenic planktonic E. coli 34. Due to the local production of urease enzyme products on their surface and substantial dispersion, U-MSNPs increase interaction with bacteria. U-MSNPs at concentrations higher than 200 μg/mL showed a remarkable 60% reduction in biofilm formation by uropathogenic E. coli strains, demonstrating highly potent antibacterial activity. Leveraging the natural catalytic pathways of enzymes, EMNMs offer a powerful platform for various biomedical interventions due to their biocompatibility and effective energy transduction. Nonetheless, despite their promise, there is a strong impetus for further research to unlock their full potential in combating bacterial infections and enhancing patient care.

### 2.3 Bio-hybrid MNMs

Some individual cells have evolved intricate mechanisms to achieve movement in low Reynolds number fluid environments. For instance, various bacterial species like E. coli, Serratia marcescens, magnetotactic bacteria, as well as sperm cells, protozoa, and algae, possess flagella or cilia that are distributed unevenly along their bodies 35. These structures enable them to generate forces for movement by propelling fluid through their environment 36-38. This unique arrangement allows for efficient locomotion and navigation in diverse biological systems. To harness this naturally occurring motility, researchers have begun integrating these unicellular entities with artificial constructs. This creates bio-hybrid MNMs by encapsulating these organisms within microtubes or tethering them to micro-scale particles. This fusion leverages the sophisticated locomotive capabilities of these cells, along with adaptable synthetic materials, to produce MNMs with high biocompatibility. These motors can potentially interact with the surrounding cells or tissues and sustain longer operational lifespans 39, 40. Consequently, this has spurred investigations into bio-hybrid MNMs as more compatible and sustainable alternatives to their fully synthetic counterparts 41, 42. Such endeavors may soon transform antimicrobial strategies and application domains.

**Microalgae**. Microalgae can provide self-propulsion through flagella beating. Bio-hybrid micromotors based on Chlamydomonas reinhardtii microalgae, have been used for cargo delivery, demonstrating efficient locomotion and good biocompatibility 43. Based on the aforementioned advantage, a bioinspired micromotors vehicle comprised of nanoparticle-modified algae has been used for actively administering antibiotics within the lungs in vivo 44. This motor is synthesized using click chemistry to attach polymer nanoparticles encapsulated with antibiotic-loaded neutrophil membranes onto natural microalgae. It propagates at a velocity exceeding 110 μm/s in simulated lung fluids and displays a uniform distribution across the inner lung tissues. In an experimental mouse model of acute pneumonia caused by Pseudomonas aeruginosa, this bio-hybrid motor effectively reduced bacterial load and considerably decreased death rates in the animal subjects. Additionally, the easy cultivation of microalgae, their sustainability over extended durations, and the convenience of modifying their surface render them particularly advantageous. Zhang et al. have used click chemistry to functionalize microalgae with angiotensin-converting enzyme 2 (ACE2) receptor against the SARS-CoV-2 spike protein 45. The ACE2-algae-robot exhibits remarkable self-propulsion, with a speed exceeding 100 μm/s, and can maintain its propulsion for over 24 hours in various aquatic environments, such as drinking water and river water. This dynamic movement enables enhanced collision, contact, and adsorption of target contaminants when compared to static counterparts. The robot's localized self-mixing effect further contributes to efficient and rapid decomposition, facilitating accelerated “on-the-fly” removal of SARS-CoV-2 spike proteins and SARS-CoV-2 pseudovirus.

**Sperm.** Motile cells with cytoskeletal molecular motors, such as sperm, have also been used to create hybrid nanomotors for various applications in biomedical research 46, aiming to achieve motion at the microscale 47. Carmen.C et al. have used high-speed aqua sperm micromotors derived from North African catfish for targeted eradication of bacteria biofilms in restricted environments 48. Their tiny size enables them to infiltrate and disrupt the biofilm structure, while their remarkable speed ensures quick and effective destruction of biofilms. This has demonstrated the successful eradication of biofilms that colonize medical and laboratory tubing.

Although these biohybrid MNMs hold great promise in future biomedical applications due to their excellent biocompatibility, minimally invasive nature, effective locomotion in low Reynolds number environments, and strong connection to biological substances, it is important to note that they are currently in the nascent phase of development. Further research is needed, particularly in the field of antimicrobial applications.

### 2.4 Chemical catalysis-driven

MNMs rely on an external power source to propel themselves in a fluid environment, and one popular method of propulsion is catalytic propulsion, especially in chemically-driven MNMs 49. These nanomotors are often made up of inorganic catalysts, such as metal nanoparticles, which could convert the chemical energy present in the surroundings into mechanical motion through chemical reactions. Chemically catalyzed MNMs play a crucial role in the antibacterial field, finding diverse applications in areas like cargo delivery 50, 51, bio-toxin detection 52, 53, and bioremediation 24. These MNMs typically rely on H_2_O_2_, water, and acid as their main fuel sources.

**H_2_O_2_**. Through decomposition, H_2_O_2_ is converted into water and oxygen gas, generating propulsion force by rapidly releasing of oxygen bubbles 54. Jurado-Sμnchez et al. have reported a Janus micromotor for bacterial endotoxin detection 53. A bottom-up approach was utilized for the synthesis of an oil-in-water emulsion, incorporating graphene quantum dots (GQDs) with a substantial quantity of platinum and iron oxide nanoparticles on a specific side of the Janus micromotor body. The consistent motion of several micromotors across a polluted sample induced consistent mixing, leading to significantly improved mass transport. Consequently, this led to an elevation in the rate of response between the lipopolysaccharide (LPS) and the micromotors present in the contaminated fluid, thereby surpassing the performance exhibited by static micromotor equivalents. Furthermore, Janus micromotors, which utilize platinum nanoparticles to catalyze bubble propulsion, are also employed as movable sensors to detect toxins secreted by enterobacteria, serving as indications of food contamination 52. They can detect endotoxin with exceptional sensitivity (0.07 ng/mL) and detect Salmonella toxin in food samples within just 15 minutes, a duration considerably shorter than the numerous hours demanded by traditional technique.

**Water/acid**. Despite H_2_O_2_'s efficient propulsion capabilities as a fuel, it does have certain drawbacks, including inherent toxicity, the possibility of tissue injury due to deleterious reactions within the body and a low fuel concentration in bodily fluids 55, 56. In lieu of this, certain MNM systems employ active metals (e.g., gallium, zinc, and magnesium) to facilitate the decomposition of acid or water, generating hydrogen gas bubbles for efficient propulsion 57-59. Jorge A et al. have synthesized Janus micromotors that are composed of magnesium microparticles serving as catalysts and utilize water propulsion 60. This novel kinetic antibacterial method demonstrates a substantial increase in antibacterial efficacy, with a notable augmentation of 27-fold when compared to its static counterpart. Furthermore, acid is employed as an alternative fuel source in addition to water. A Janus Ga/Zn micromotor has been created by unevenly depositing liquid metal Ga on Zn microparticles for the treatment of bacterial infections 61. The propulsion mechanism of this system involves the generation of hydrogen gas bubbles through the zinc-acid interaction. In simulated stomach acid conditions, the system demonstrates self-propulsion, achieving a maximum speed of 383 millimeters per second. The movement exhibited by the Janus Ga/Zn micromotor facilitates the dispersion of Ga, resulting in a significant enhancement in the antibacterial efficacy against H. pylori in comparison to stationary Ga microparticles.

Chemically propelled nanomotors have broad applications in biomedical, environmental, and hygiene fields. However, they still face challenges such as high dependence on chemical environments, instability of catalysts, and low biocompatibility. Therefore, further research and development are needed to address these issues and optimize their performance.

## 3. Antibacterial effect of micro/nanomotors

### 3.1 Bactericidal action

#### 3.1.1 Drug delivery

The intersection of MNMs with the field of medication delivery marked a groundbreaking development in the early 2000s 62. Compared to conventional systems of passive drug delivery, actively propelled drug delivery platforms utilizing highly self-propelled nanomotors offer a multitude of advantages. For instance, their nanoscale-specific surface area significantly amplifies the drug loading capacity 63, 64, which offers protection for the drugs, thereby reducing potential toxicity and side effects 65, 66. Moreover, these platforms enable sustained release profiles over a specified timeframe 67, permit targeted therapy 64, and facilitate precise delivery and deep penetration within tissues. These unique characteristics make them well-suited candidates for innovative drug delivery approaches and are extensively utilized in the realm of antibacterial treatment 68. Their applications range from the eroding of stubborn biofilms, which presents a significant challenge in clinical settings, to the treatment of deep-seated bacterial infections in the human body, as well as the restoration of impaired biological environments.

**Figure 2:**
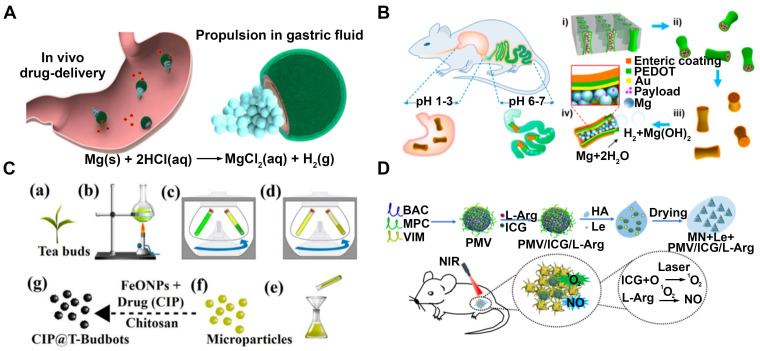
MNMs for drug delivery. **(A)** In vivo propulsion and drug delivery of the Mg-based micromotors in stomach. Adapted with permission from 51, copyright 2017 Springer Nature.** (B)** EMgMs for localized drug delivery to the GI tract. Adapted with permission from 69, copyright 2016 American Chemical Society. **(C)** Fabrication of CIP loaded T-Budbots for biofilm eradication. Adapted with permission from 22, copyright 2020 American Chemical Society.** (D)** The microneedle patches used for luteolin delivery in wound antibiofilm therapy. Adapted with permission from 75, copyright 2023 Elsevier.

**Treatment of bacterial infections in vivo**. Drug delivery vehicles used in vivo need to maintain good biocompatibility, avoid clearance by the immune system and exhibit excellent tissue targeting. Ávila's group has developed an efficient treatment for stomach infections using magnesium-based micromotors 51. These micromotors consist of a poly (lactic-co-glycolic acid) (PLGA) layer loaded with Clarithromycin (CLR) and a layer of chitosan polymer encapsulating a propellant magnesium center (**Figure 2A**). The micromotors demonstrated the ability to propel themselves within the gastric fluid and the presence of a chitosan layer notably enhanced their capacity to adhere to and remain attached to the stomach wall. This allows CLR to be locally and autonomously released from the PLGA polymer coating, facilitating effective treatment. Furthermore, some magnesium-based tubular micromotors 69, coated with an enteric polymer layer, have been developed as a reliable nanobiotechnology tool for targeted gastrointestinal (GI) delivery (**Figure 2B**). Upon activation, the aforementioned motors autonomously navigate through the stomach fluid, thereby initiating their operation within the GI tract. Subsequently, they successfully enter the intended tissue and persist within it to discharge their designated payloads.

Additionally, human blood cells such as platelets are also used to build nanomotors for drug delivery. Tang et al. have developed a urease-powered Janus platelet micromotor, which provides a range of functions in response to different situations such as hemostasis, inflammation, and wound healing 31. Platelets possess versatile receptors on their surface that allow them to specifically bind to biological threats like bacteria. These JPL-motors demonstrate efficient propulsion when exposed to urea fuel, significantly improving their attachment effectiveness with the targeted biological entities. When the motors are loaded with antimicrobial medications, they demonstrate an increased effectiveness in therapy.

**Biofilm eradication**. Formation of biofilms is a strategy adopted by microorganisms to adapt to specific environmental conditions. Biofilms composed of complex microbial communities and extracellular polymeric substances, which include proteins and polysaccharides, are typically the source of bacterial infections. However, these extracellular polymeric substances pose a challenge to the effective penetration of small-molecule antibiotics through the biofilm structure 70. Compared to free-floating bacteria, bacteria residing within biofilms exhibit formidable levels of antibiotic resistance, as they can withstand concentrations up to 1000 times higher 71, 72, making them exceptionally difficult to treat and eradicate 73, 74. In such scenarios, nanomotors have shown remarkable drug delivery capabilities and have started to be utilized for the eradication and treatment of biofilms.

Bhuyan et al. have developed magnetotactic microbots, known as T-Budbots 22, which are created from mesoporous tea buds of Camellia sinensis and are equipped with Ciprofloxacin (CIP) incorporated into their porous structure (**Figure 2C**). These microbots exhibit the ability to be magnetically guided along the biofilm matrix, allowing them to precisely remove or break down biofilms and enhance the penetration of antibiotics into the biofilm. These microbots offer a drug delivery method that demonstrates high efficiency in releasing drugs in a pH-dependent manner, hence assuring sustained release within the acidic milieu commonly found in biofilms. Nanomotors have also been used to load antibacterial substances such as luteolin for the treatment of biofilm-related infections in wounds (**Figure 2D**) 75. These nanomotors are equipped with microneedles that can physically penetrate biofilms. Upon release, luteolin exhibits a strong antibacterial effect by damaging bacterial cell membranes and also inhibits biofilm formation. This approach shows promise in effectively treating bacterial biofilm-related infections and promoting wound healing.

**Environmental remediation**. As drug delivery systems, Nanomotors have advantages such as biodegradability, environmental friendliness and low toxicity. In the field of environmental remediation, especially in water pollution control, they have the belief in potential for application. Jorge A et al. have recently adopted a chitosan-based micromotor for the treatment of contaminated water samples that include unidentifiable bacteria 60. These Janus micromotors consist of magnesium microparticles coated with the biodegradable and biocompatible polymers PLGA, alginate (Alg), and chitosan (Chi) (**Figure 2E**). In various controlled experiments involving the treatment of drinking water contaminated with E. coli bacteria, these dynamic micromotors yields killing effectiveness of 96% within 10 minutes, compared with the static counterpart with only a 4% killing efficiency, reaching up to a 27-fold improvement antibacterial efficiency. The water-powered nanomotor, characterized by its favorable biocompatibility, exhibits significant potential for a wide range of applications, including the eradication of bacteria in water and environmental remediation.

#### 3.1.2 Metal ions

Metal nanoparticles can generate hydrogen bubbles by catalyzing water or acid or through ionic diffusionphoresis to produce strong propulsion for nanomotors. In addition, metal ions possess inherent antibacterial activity and have been utilized to treat injuries and ulcers 76. Taking silver ions as an example, silver ions can bind to proteins and DNA within the bacterial cell membrane, thus causing disruptions to bacterial metabolism and growth. Additionally, the presence of silver ions has the potential to break the structural integrity of the bacterial cell wall, leading to the subsequent demise of the bacterium. Based on these properties, metal nanoparticle nanomotors with high biocompatibility, environmental friendliness, and efficient movement capability hold great potential in the field of antibacterial applications.

**Figure 3:**
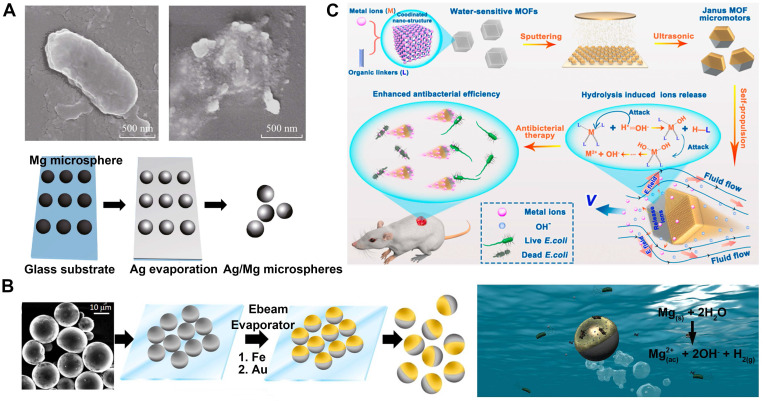
MNMs for antibacterial with metal ions.** (A)** Ag/Mg bactericidal micromotor. Adapted with permission from 77, copyright 2015 Springer Nature. **(B)** AgNP-coated Janus microbots for killing bacteria in water. Adapted with permission from 24, copyright 2017 American Chemical Society. **(C)** The fabrication process of the MOF micromotors and their antibacterial wound therapy. Adapted with permission from 78, copyright 2022 American Chemical Society.

Ge et al. have successfully fabricated a Janus micromotor using a magnesium microsphere coated with silver on a specific side through the thermal evaporation method (**Figure 3A**) 77. The increased silver ion emission from the mobile micromotor in comparison to the stationary one is responsible for the micromotor's enhanced antibacterial properties. Facilitated by the motion-based solution mixing procedure, the dissolved silver ions reach the bacteria more rapidly, resulting in a remarkable nine-fold increase in bacterial eradication compared to the static micromotor. The utilization of microbots adorned with silver nanoparticles (**Figure 3B**), employed by Vilela's research group 24, has been used to disinfect Escherichia coli bacteria and their subsequent elimination from polluted water supplies. This study revealed that these microbots exhibit remarkable efficiency in killing E. coli, with a success rate exceeding 80% in just 15 minutes of exposure to contaminated water solutions.

Metal organic framework (MOF) micromotors (**Figure 3C**) are capable of swimming through a medium with a very efficient conversion of energy, which is achieved by the utilization of ion gradients that arise from the spontaneous breakdown of the MOF structure 78. The exposed surfaces of the MOF particles are covered by a coating layer (e.g., Au, Ag, Pt or Ni) with thermal evaporation to prepare the Janus structure. The uncoated side of the Janus MOF micromotor would release ions gradually on account of the MOFs' sensitivity to water. The antibacterial mechanisms of MOFs are believed to be comparable to those of metal ions due to the release of metal ions. These mechanisms include the generation of reactive oxygen species (ROS), alteration of ion equilibrium, destruction of cell walls, interaction with protein thiol groups, inactivation of crucial enzymes, and infliction of cell wall harm 79. In addition to their efficient propulsion, the metal cations that are produced by the MOF micromotors possess antibacterial properties, which enables them to effectively eliminate Escherichia coli bacteria, benefiting from their heightened efficiency due to their motion. By utilizing this dynamic antibacterial strategy, MOF micromotors can serve as effective agents for combating bacterial infections.

#### 3.1.3 Photothermal therapy (PTT)/ photodynamic therapy (PDT)

**Figure 4:**
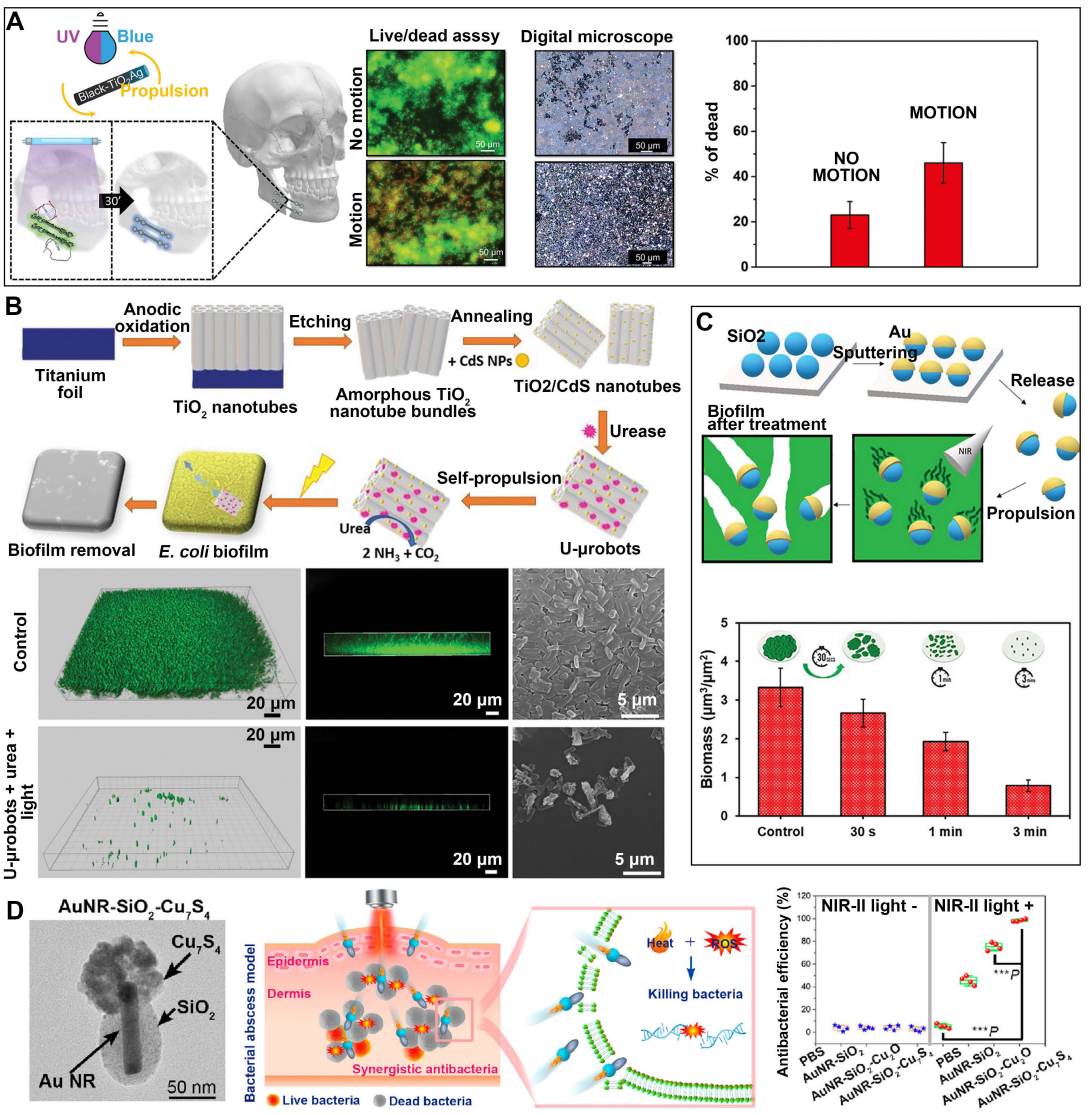
MNMs for antibacterial photodynamic therapy or photothermal therapy. **(A)** Light-driven self-propelled tubular B-TiO_2_/Ag nanorobots to remove multispecies biofilm from facial titanium miniplates. Adapted with permission from 81, copyright 2022 WILEY. **(B)** Enzyme-photocatalyst tandem microrobot for Escherichia coli biofilm eradication. Adapted with permission from 32, copyright 2022 WILEY. **(C)** Mesoporous SiO_2_/Au nanomotors for biofilm eradication. Adapted with permission from 11, copyright 2023 WILEY. **(D)** NIR-II light-driven dual plasmonic (AuNR-SiO2-Cu_7_S_4_) antimicrobial nanomotors synergistic photothermal and photocatalytic treatment of bacterial infections. Adapted with permission from 12, copyright 2023 American Chemical Society.

**PDT**. The antimicrobial action of PDT is rooted in the activation of a photosensitizer by light, which triggers the generation of ROS that indiscriminately eliminates bacteria through an oxidative burst 80. In recent years, scientists have combined the self-directed locomotion of nanomotors with the antimicrobial properties of PDT by loading photosensitizers onto nanomotors, opening new avenues for antibacterial approaches. Ussia et al. have developed tubular black-TiO_2_/Ag nanorobots propelled by light (**Figure 4A**), which can effectively treat infections caused by biofilm on metallic miniplates 81. These nanorobots exhibit enhanced antibacterial activity by releasing increased levels of ROS and Ag ions when exposed to light. Moreover, Villa's group has developed a hybrid enzyme/photocatalytic microrobot using urease-immobilized TiO_2_/CdS nanotube bundles 32, which has shown promise in eradicating Escherichia coli biofilm (**Figure 4B**). Upon exposure to visible light, the photocatalytic element of the microrobot initiates the production of reactive radicals, which subsequently produce a phototoxic impact on the surface of biofilm. After a 2-hour illumination, these microrobots successfully eliminate nearly 90% of the bacterial biofilm.

**PTT**. In recent years, PTT has garnered significant attention due to its remarkable ability to eliminate bacteria without inducing drug resistance 82. In the process of PTT, photothermal agents harness absorbed light to generate heat, resulting in membrane rupture, protein denaturation, and ultimately the irreversible death of pathogenic bacteria 83. In a manner akin to photodynamic therapy, the utilization of nanomotors in conjunction with PTT has been employed to elicit antibacterial properties. Loukanov et al. have developed Janus nanomotors featuring a gold nanolayer coating, capable of generating heat upon NIR irradiation to achieve photothermal lysis of Escherichia coli O157:H7 10. Furthermore, nanomotors made by coating mesoporous-silica nanoparticles with a thin gold layer have also been developed for the eradication of Pseudomonas aeruginosa biofilm 11. The self-propulsion of mesoporous SiO_2_-Au nanomotors is effectively shown with the application of near-infrared light. Thus, the nanomotors exhibit the capability to effectively permeate the biofilm matrix and disseminate the biofilm within its initial position by means of the photothermal action induced by the gold constituent (**Figure 4C**).

**PTT+PDT**. Bringing together PDT and PTT directly has been observed in several experimental studies to yield a synergistic enhancement of antibiofilm and bactericidal effects 84. PTT facilitates the augmented intracellular permeation of reactive oxygen species generated by PDT through the heat-induced enhancement of bacterial membrane permeability 85. Moreover, the combination of PTT with PDT can reduce the required laser intensity in PTT, thereby minimizing the risk of overheating and injury to normal tissues 86. Liu et al. have successfully fabricated a Janus nanomotor with a matchstick-like morphology, referred to as Au@ZnO@SiO_2_-ICG nanomotor, for light-responsive combinatorial antibacterial treatment 87. The inclusion of ICG as a photosensitizer augments the photothermal effect, particularly when jointly employed with Au under near-infrared illumination. Additionally, when exposed to ultraviolet radiation, the metal/semiconductor heterostructure of Au@ZnO enhances the generation of cytotoxic reactive oxygen species for photodynamic sterilization. The findings demonstrate the exceptional light responsiveness and synergistic sterilization capabilities of the prepared Au@ZnO@SiO_2_-ICG nanomotors against both Gram-negative E. coli and Gram-positive Staphylococcus aureus (S. aureus) bacteria. A recent study has documented the development of dual plasmonic antimicrobial nanomotors with a Janus structure, powered by NIR-II light 12. These nanomotors, composed of AuNR-SiO_2_-Cu_7_S_4_, exhibit the ability to effectively cure bacterial infections that are resistant to many drugs. The nanomotors exhibit significant enhancements in both photothermal performance and photocatalytic activity due to the robust plasmon coupling between AuNRs and the Cu_7_S_4_ component, as well as the augmented energy transfer. The locomotion characteristics of nanomotors facilitate the process of transdermal penetration and augment the interaction with bacteria (**Figure 4D**).

#### 3.1.5 Chemodynamic therapy (CDT)

**Figure 5:**
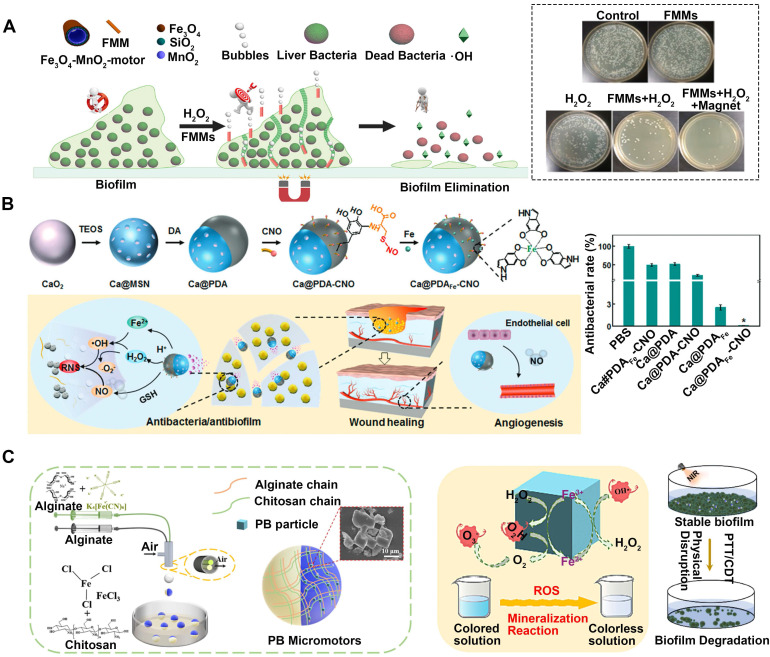
MNMs for antibacterial chemodynamic therapy.** (A)** Multifunctional motors with enhanced antibacterial activity against refractory biofilm infections. Adapted with permission from 93, copyright 2022 Elsevier.** (B)** Janus Ca@PDAFe-CNO nanomotors integrated with biofilm microenvironment for effective sterilization. Adapted with permission from 3, copyright 2022 WILEY. **(C)** A self-propelled Prussian blue micromotor combinate with PTT, CDT for achieving physically and chemically disrupt stable biofilm. Adapted with permission from 94, copyright 2022 Elsevier.

CDT is driven by the Fenton reaction 88, which catalytically generates highly toxic ROS from H_2_O_2_ through Fe^2+^/Fe^3+^ Fenton reactions 89. ROS can inactivate various pathogens through mechanisms such as protein dysfunction, impaired membrane function, and interference with nutrient assimilation 90-92. However, ROS suffers certain limitations including short diffusion distances, susceptibility to stability issues as well as difficulties in direct transport to infection sites. Ji et al. have designed a magnetic catheter micromotor for the treatment of bacterial biofilm infections 93, which adopts H_2_O_2_ for the fuel and MnO_2_ to serve as the catalyst. Additionally, ferromagnetic nanoparticles (MNPs, Fe_3_O_4_) are incorporated onto the outer surface of the micromotor (**Figure 5A**). These remotely controlled motors can infiltrate the extracellular polymeric substance of the biofilm and effectively break it by levering the aid of bubbles. Moreover, the Fe_3_O_4_ nanoparticles facilitate the conversion of H_2_O_2_ into highly poisonous •OH, which successfully eradicates the vulnerable bacteria. This innovative strategy integrates mechanical damage, the production of highly toxic •OH, and precise magnetic guidance into a unified system, which efficiently eliminates biologically contagious fouling within microchannels in just 10 minutes. In addition, CaO_2_ nanoparticles are capped with polydopamine (PDA) layers, followed by complexation with Fe^2+^ and surface grafting of cysteine-NO (**Figure 5B**) to counter biofilm infections and expedite angiogenesis and wound healing without relying on antibiotics 3. The Fenton reaction converts the emitted H_2_O_2_ and Fe^2+^ into •OH in response to the low pH in the biofilm microenvironment, which effectively destroys biofilms and eradicates the bacteria residing within them, both in laboratory settings (in vitro) and living organisms (in vivo).

In addition to the above metals, some special substances can also be used for CDT. For example, Prussian blue (PB) can catalyze the reduction of H_2_O_2_ to ROS, which is hazardous to living organisms. Additionally, PB exhibits excellent photothermal conversion capabilities in the NIR region, making it an ideal photothermal agent for bacteria ablation. Thus, a self-propelled system of Prussian blue micromotors (PB MMs) was used in conjunction with CDT and PTT to efficiently degrade biofilms (**Figure 5C**) 94. This multifunctional system acts as a therapeutic agent, chemically killing bacteria and physically destroying biofilms.

### 3.2 Detection

Conventional methods for bacterial detection, like culture-based techniques and PCR, demand complex equipment 95, as well as the need for specialized personnel and extended processing times 96; scientists have been dedicated to developing more sensitive, efficient, and rapid bacterial detection techniques. Traditional fluorescent dyes and nanoparticles, such as QDs, have been employed in the design of MNMs for the detection of chemical weapons and heavy metal threats. These MNMs allow for real-time fluorescence visualization and analysis, facilitating the identification of hazardous substances 52, 97. Furthermore, researchers have engineered MNMs to function as autonomous sensing devices capable of detecting pathogens or toxins with high specificity.

**Figure 6:**
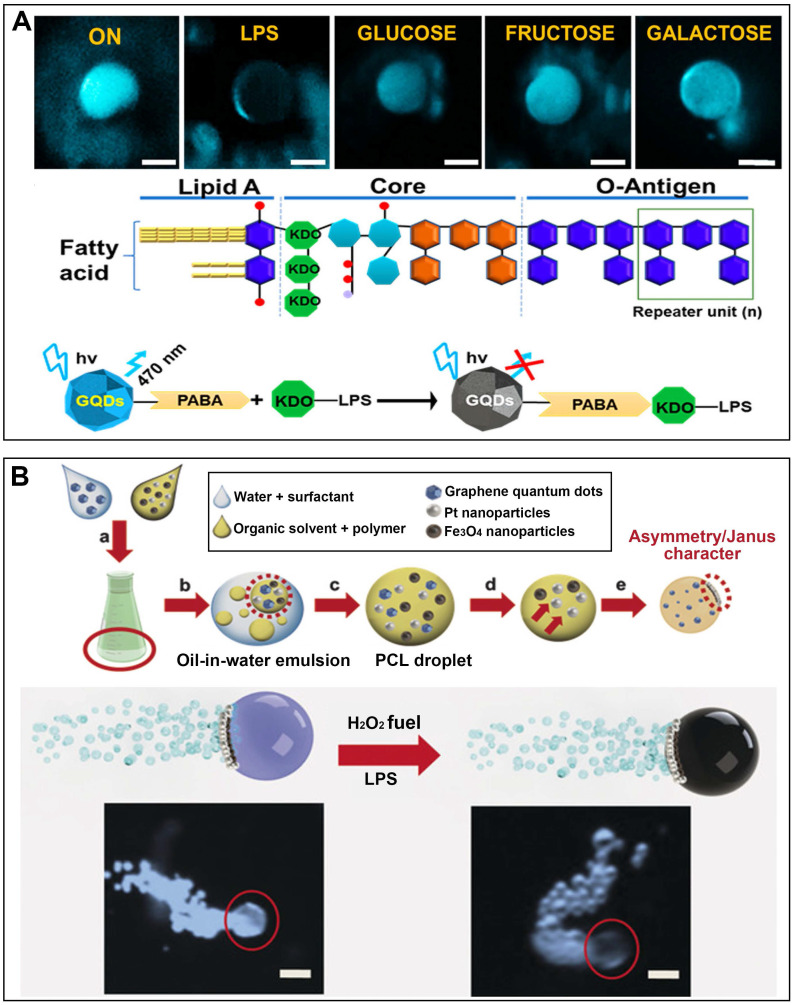
MNMs for detection. **(A)** Self-propelled Janus microsensors for the detection LPS from Salmonella enterica. Adapted with permission from 52, copyright 2018 American Chemical Society.** (B)** Magneto-catalytic graphene QDs based Janus micromotors for bacterial endotoxin detection. Adapted with permission from 53, copyright 2017 WILEY.

Pacheco et al. have reported on the utilization of Janus micromotors as mobile sensors for detecting toxins released by enterobacteria 52. These micromotors are fabricated using a pickering emulsion method and employ the simultaneous encapsulation of platinum nanoparticles to enhance bubble propulsion, along with receptor-functionalized QDs (**Figure 6A**). As the main indicator for enterobacteria, Salmonella enterica, interacts with the QDs, the native fluorescence of the micromotors is rapidly quenched in a concentration-dependent manner. Magnetocatalytic hybrid micromotor has been developed to wrap PABA-GQDs for the purpose of detecting bacteria endotoxins (**Figure 6B**) 53. The presence of PABA tags on the micromotor enables highly specific recognition of LPS. Conversely, the interaction between the GQDs and target endotoxin leads to the suppression of fluorescence. This innovative fluorescent detection platform based on micromotors shows great potential for detecting a wide range of chemical toxins.

In the detection system of MNMs, fluorescent materials with aggregation-induced emission (AIE) characteristics, such as tetraphenylethene (TPE) and its derivatives, are alternatively employed 33. The interaction between MNMs and E. coli is responsible for the AIE effect of TPE derivatives, leading to the enhanced fluorescence emission of MNMs, which can be easily detected by direct visual observation. The fluorescence strength is associated with the amount of caught bacteria and the bacterial quantity in the suspensions. Consequently, compared to their stationary counterparts, MNMs are expected to enhance the sensitivity and reliability of E. coli monitoring.

### 3.3 Capture and isolation

By functionalizing with various capturing agents such as specific antigens or cell receptors, MNMs enable target-specific binding through an "on-the-fly" behavior 98. Compared to traditional passive diffusion capturing agents, MNMs enhance the efficiency of capture and transportation through their unique autonomous motion, making them suitable for the capture and removal of microorganisms or detoxification.

**Figure 7:**
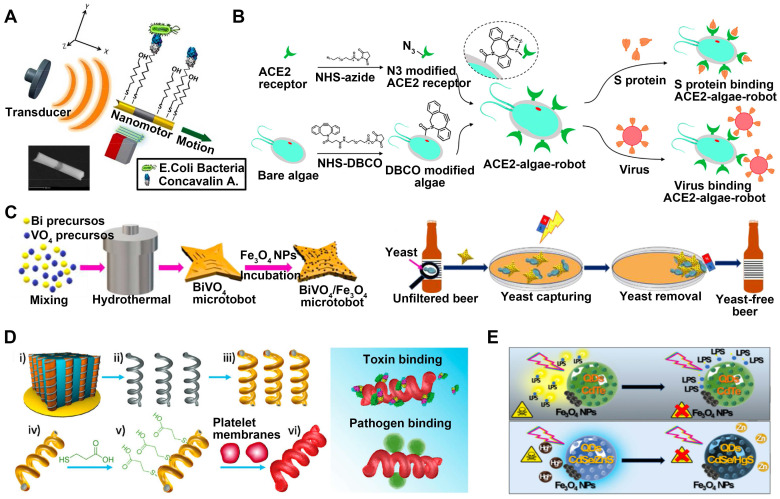
MNMs for capture and isolation.** (A)** Ultrasound-propelled magnetically-guided receptor-functionalized nanowire motor for selective capture and transport of biological targets. Adapted with permission from 23, copyright 2013 American Chemical Society. **(B)** ACE2-algae-microrobot for the binding and removal of spike protein and SARS-CoV-2 virus. Adapted with permission from 45, copyright 2021 American Chemical Society. **(C)** Dual magnetic/light-powered hybrid microrobots for removal of yeast cells in beer. Adapted with permission from 99, copyright 2020 WILEY. **(D)** PL-motors for binding and isolation of platelet-specific toxins and pathogens. Adapted with permission from 21, copyright 2017 WILEY. **(E)** QDs/Fe_3_O_4_ light driven micromotors for detoxification. Adapted with permission from 15, copyright 2019 WILEY.

**Microorganism**. Garcia-Gradilla et al. developed nanowire motors that are guided by magnets and powered by ultrasound (**Figure 7A**) 23. These motors are functionalized with lectin and anti-protein A antibody bioreceptors, enabling the capture and transportation of E. coli and S. aureus bacteria, respectively. Incorporating a nickel segment allows for magnetically-guided motion and enables the transportation of substantial cargo following predetermined pathways. The resulting nanomachine demonstrates the ability to target and transport various cargo in a controlled and cooperative manner without being constrained by its environment or causing harm to biological substances. Additionally, researchers have developed microrobots based on algae that have been modified with ACE2 receptors 45, enabling the effective elimination of SARS-CoV-2 from polluted water reservoirs (**Figure 7B**). The ACE2 receptor exhibits a strong affinity for the S1 subunit of the viral spike protein, allowing for adequate recognition of the target virus. The ACE2-algae robot demonstrates fast and enduring self-propulsion in diverse aquatic settings. Regarding fungi, once activated by light, the BiVO_4_ micromotors (**Figure 7C**) displayed the capability to actively locate and attach to yeast cell walls 99. When introduced into an unfiltered beer sample, these micromachines successfully eliminated nearly 100% of residual yeasts. Furthermore, it is worth noting that they have the potential to be included in the fermentation process from its inception without any discernible impact on key beer attributes such as alcohol concentration or coloration. This finding implies that externally controlled microrobots can serve as a cost-effective and inventive measure for mitigating yeast contamination in complex liquid settings.

**Detoxification**. By combining toxin adsorption and self-movement ability, MNMs primarily accomplish their detoxification function. Li et al. have prepared a biomimetic nanorobot by utilizing magnetic helical nanomotors covered with the plasma membrane (**Figure 7D**) 21. This innovative nanorobot is designed to adsorb and isolate platelet-targeted biological agents. In experimental trials using a Vero cell assay, it has been demonstrated that these platelet-mimicking motors effectively adsorb Shiga toxin (Stx) through robust binding facilitated by the presence of receptors on the platelet surface. The biomimetic nanomotors possess motility capabilities and exhibit platelet-like biological properties, which make them very promising for effectively binding and isolating various biological threats.

In a study conducted by Pacheco's research group 15, 16, they have introduced a micromotor composed of degradable polymers such as PCL and PLGA, which are encapsulated with CdSe@ZnS QDs and asymmetric Fe_3_O_4_ NPs patch (**Figure 7E**). The micromotor has proficient ability in navigating and detoxifying blood samples. Through electrostatic and hydrophobic binding mechanisms, the polymeric layer of the micromotor can effectively adsorb LPS, resulting in the successful elimination of E. coli O111:B4 toxin from the bloodstream.

## 4. Challenges and outlook

Previously, we presented a synthesis of the current state of research regarding MNMs for antibacterial use. It is incontrovertible that MNMs hold groundbreaking importance in the sphere of antimicrobial interventions. Powered by external energy sources, catalytic enzyme reactions, and other mechanisms, MNMs are endowed with remarkable propulsive forces that grant them exceptional mobility. This dynamic movement substantially enhances their ability to breach bacterial biofilms, addressing a critical limitation of traditional antimicrobial agents. In aqueous environments, their heightened mobility significantly increases interactions and adherence with bacterial targets. The integration of antimicrobial drugs within MNMs markedly improves their antibacterial efficacy through active transport methods, contrasting the less effective passive diffusion. In detection applications, the advent of MNM-based detection platforms offers a promising alternative to overcome the drawbacks of conventional methods. Equipped with targeted receptors, MNMs pioneer a detection method that not only conserves time but also sidesteps the necessity for complex, precise instrumentation, potentially enabling instantaneous pathogen monitoring.

However, current research is limited to the laboratory stage, and the process of clinical translation continues to encounter obstacles. Initially, it is imperative that the constituents employed in the fabrication of MNMs do not possess any adverse effects on the human organism. Furthermore, MNMs should have good biocompatibility and be able to evade the body's immune system attacks and clearance, otherwise their effectiveness will be affected. At the same time, the issue of insufficient propulsion of MNMs within the body needs to be addressed. Currently, fuels such as H_2_O_2_ used to propel MNMs not only pose certain risks to the human body but also have considerably lower concentrations in the biological organism compared to the laboratory, thereby impeding the efficient propulsion of MNMs. Other external energy sources, such as ultrasound and magnetic fields, also have certain usage limitations. Therefore, the clinical translation of MNMs for antimicrobial purposes still faces difficulties. Meanwhile, we proceeded to discuss the potential outlook for other existing challenges in the future.

### 4.1 Controlled drug release at specific sites within the body using MNMs

The biggest challenge in using MNMs as drug delivery carriers in the body is how to ensure their release of drugs, specifically at the designated bacterial infection site. It is well-known that antibiotic usage within the human body can disrupt the native microbiota, such as causing diarrhea and constipation when affecting the gastrointestinal microbiota. It can also kill beneficial bacteria, leading to immune system imbalance and increased risk of infection. Moreover, antibiotics can exhibit toxicity to the liver and kidneys. Therefore, it is crucial to ensure that MNMs release antibiotics at the specific site of bacterial infection within the body while minimizing any effects on other organs and systems. There are two issues involved:

**The targeting ability of MNMs towards bacterial infection sites**. Based on current research, scientists have utilized neutrophil 44, or platelet cell membranes to encapsulate nanoparticles and construct MNMs 31. Through the intrinsic inflammatory chemotaxis of these blood cells, these MNMs can achieve targeted action against bacteria and inflammatory mediators released during infection. However, using live cells to construct MNMs may face challenges such as difficulty sourcing cells and issues of immune rejection between different organisms. Other researchers have loaded chitosan onto MNMs to achieve targeted treatment of Helicobacter pylori infection in the stomach, utilizing chitosan's high affinity for gastric epithelial cells 51. However, the adhesion strength of chitosan mainly relies on charge affinity and the action of adherent molecules, which are influenced by various factors and exhibit individual variability. Additionally, it lacks specific targeting towards gastric epithelial cells and is prone to instability. Therefore, future development of MNMs should focus on improving their targeting ability towards infected tissues and organs within the body to minimize adverse effects on the body.

**Controlled drug release for MNMs in vivo**. It is important to avoid premature drug release before reaching the designated location. If MNMs release drugs at any time during transport, it will inevitably cause harm to other tissues and organs of the body and lose the advantage of their active mobility. Currently, scientists have attained manipulation over the carrying and unloading of cargo in MNMs using a “light shift” method 14, but there are issues such as the harmful effects of ultraviolet light on the body and the insufficient tissue penetration ability of visible light to reach the interior of the body for controlling MNMs. Other scientists have used thermally responsive polymers to construct MNMs, achieving controlled drug release during heat treatment 77, but the heat generated may still be damaging to the tissue structure of the body, which requires further exploration. Based on the existing issues, the future study and development of MNMs should prioritize the investigation of strategies to attain precise control over the discharge of their contents. Our idea is to leverage the specificity of different tissue and organ structures or environments in the body, such as differences in physicochemical properties, the presence of specific cells in tissue structures, or by using external physical methods with sufficient penetration and no harm to the body to achieve controlled drug release for MNMs.

### 4.2 Combined effects of multiple bactericidal mechanisms based on MNMs

Despite the significant improvement in the efficiency and effectiveness of various bactericidal mechanisms with the assistance of MNMs' mechanical motion capabilities, there are still some issues. For example, the gradual increase in bacterial resistance may render the antibiotics delivered by existing MNMs insufficient to kill bacteria or higher dosages may be required to achieve satisfactory results. The heat generated by the photothermal therapy utilized by MNMs can inevitably cause damage to surrounding tissues. The ROS generated during CDT and PDT can damage the DNA, protein, and other surrounding normal cells. Additionally, this process can trigger the activation of inflammatory cells and give rise to localized inflammatory reactions, resulting in pain and discomfort for patients. Therefore, we propose the combined application of multiple bactericidal mechanisms based on MNMs by developing and researching the inherent framework structure of MNMs or the loaded nanoparticles. Moreover, the design and proportion of each bactericidal mechanism's effects are considered, aiming to maximize bactericidal performance while minimizing side effects.

### 4.3 Improve the accuracy, sensitivity and convenience of MNMs detection methods

The MNMs-based bacterial or toxin detection platform is achieved by functionalizing receptors loaded onto MNMs, with the assistance of MNMs' autonomous movement capability, to achieve “on-the-fly” binding with bacteria or toxins. This detection method eliminates the need for sample preparation, enables real-time detection, significantly shortens the detection time compared to traditional methods and improves detection efficiency. Despite its significant advantages, further development and research are still required. In terms of accuracy, scientists currently utilize LPS receptors loaded on MNMs for specific detection of endotoxins 53. Nevertheless, the current method only confirms the presence of endotoxins and does not have the capability to detect specific bacterial antigens. Endotoxins are found in the cell walls of most Gram-negative bacteria, such as Salmonella, Shigella and various other species. As a result, these MNMs for endotoxin detection cannot distinguish between different types of Gram-negative bacteria. To address this limitation, we propose that the MNMs detection platform should enhance its accuracy by incorporating specific bacterial antigen antibodies. This modification would enable the detection of specific bacteria and the differentiation between bacterial species. Therefore, in the future, the use of different specific bacterial antigen antibodies can be explored to improve MNMs for precise detection of different bacteria and develop multi-detection platforms. Furthermore, to enhance the sensitivity of the MNMs detection platform, the focus should primarily be on detecting ultra-trace toxins. Ultra-trace analysis is of great significance in environmental monitoring, food safety, medical diagnosis, and other fields. Even at extremely low concentrations, certain toxins can cause significant harm to the environment or organisms, posing potential biological threats. Future advancements in the MNMs detection platform could focus on the detection of ultra-trace toxins. Enhancements could include the utilization of more sensitive QDs or fluorescent markers to improve MNMs, loading a greater number of detection markers without compromising the performance of MNMs, and refining the structural morphology of MNMs to bolster their toxin-capturing capabilities. Such improvements would significantly increase the sensitivity of the MNMs detection platform. Efforts can also be directed toward integrating the MNMs detection platform with compact, user-friendly devices. This could involve the synergy of smartphones, smartwatches, or other personal electronic gadgets for real-time monitoring. By eliminating the dependence on bulky detection apparatus, this integration would facilitate the substantial enhancement of efficiency and convenience, paving the way for on-the-go environmental or health diagnostics.

### 4.4 The improvement of antibacterial MNMs propulsion system and structural design

The current generation of MNMs typically relies on a singular propulsion method for autonomous movement, which is constrained in speed and may not be effective for tasks such as bacteria capture in liquids or penetration of biofilms. In particular, enzyme-powered MNMs often require high substrate fuel concentrations to achieve the necessary energy levels, an approach that is less suitable for in-vivo applications and could lead to environmental contamination. To enhance their efficacy, particularly within the realm of antibacterial applications, there is a pressing need to engineer MNMs with multi-modal propulsion systems that can operate at greater velocities. One strategy is to augment the materials loaded onto the MNMs so that they possess an array of propulsion mechanisms concurrently. Another tactic involves the refinement of the MNMs' carrier material itself, imbuing it with intrinsic propulsion qualities. This adjustment would reduce the need for additional functional components, potentially lightening the load and increasing the MNMs' speed. Such innovations are crucial for expanding the practicality and application range of MNMs, especially in complex biological and environmental settings.

Furthermore, when using MNMs to remove biofilms to combat bacterial infections, it is crucial to consider the impact of different shapes of MNMs on their antibacterial efficacy. For example, spherical or semi-spherical MNMs experience greater resistance when penetrating biological membranes, while tubular or needle-shaped MNMs encounter less resistance, making it easier to penetrate and exert their antibacterial effects 93. Additionally, bacterial biofilms often form in confined geometric structures or small areas such as tubes, respirators, and implanted medical devices. Therefore, MNMs that are either overly bulky or excessively rigid are ill-equipped to navigate and deliver treatment within such confined spaces. To address these challenges, there is a compelling argument for future MNM research to focus on downsizing MNMs and introducing structural flexibility into their design. By taking cues from cellular biology, for instance, the integration of elements mimicking the cytoskeletal structures of cells into MNMs could confer upon them a dynamic shape-altering ability akin to that of erythrocytes (red blood cells). This transformative capability would empower MNMs with a remarkable proficiency to course through complex environments, facilitating access to targeted areas, ensuring consistent delivery of therapeutic effects, and ultimately preserving their operational efficiency.

## 5. Conclusion

This article provides an overview of the propulsion systems of MNMs used in the field of antibacterial research, as well as the latest research advancements in the application of MNMs for sterilization, capture, and detection (**Table 1**). Despite MNMs' potential to revolutionize antibacterial research, the translation from laboratory to clinical use remains fraught with challenges. Based on the current research status, we summarized the potential challenges and possible solutions and have also prospected several key directions for future studies of MNMs for antimicrobial purposes. In summary, the potential impact of nanomotors on antibacterial research is expected to be significant, offering several opportunities for advancement as the technology continues to develop and improve.

## Figures and Tables

**Table 1:** Summary of the function, mechanism, materials of antibacterial MNMs.

Function	Mechanism	Agent	Propulsion system	Materials	Ref
**Bactericidal action**	Drug delivery	Antibiotic	Bio-hybrid	Microalgae	44
		Antibiotic	Enzyme	Urease	31
		Antibiotic	Bio-hybrid	Neutrophils	11
		Antibiotic	Chemical catalysis	Mg-H_2_O	69
		Clarithromycin	Chemical catalysis	Mg-acid	51
		Clarithromycin	Chemical catalysis	Pt-acid	5
		Erythromycin	Chemical catalysis	Pt-H_2_O_2_	12
		Ciprofloxacin	magnetic	Tea buds	22
		Chitosan	Chemical catalysis	Mg-H_2_O	60
		Lysozyme	Ultrasound	Au	28
		Amps	Enzyme	Urease	30
		Nisin	Chemical catalysis magnetic	PtNPs-H_2_O_2_ Fe_2_O_3_(magnetic)	31
	PDT	TiO_2_/CdS	Enzyme	Urease	32
		Black TiO_2_	Light	Black TiO2	81
		BiVO_4_	Light magnetic	BiVO_4_-VIS Fe_3_O_4_	46
		TiO2	Chemical catalysis-	TiO2-H_2_O_2_	47
	PTT	Au	Light	Au-NIR	10
		AuNR-SiO_2_-Cu_7_S_4_	Light	AuNR-Cu_7_S_4_-NIR	12
	Metal ions	Ag	Chemical catalysis-	Ag\Mg-H_2_O\H_2_O_2_	77
		Ag	Chemical catalysis- magnetic	Mg-H_2_O Fe	24
		Ga	Chemical catalysis-	Zn-acid	61
	CDT	Fe_3_O_4_	Chemical catalysis- magnetic	MnO_2_-H_2_O_2_ Fe_3_O_4_	93
	PDT+PTT	Au/ZnO/SiO_2_-ICG	Light	Au\ICG-NIR	51
	CDT+PTT	Prussian blue	Chemical catalysis-	Prussian blue	94
	PTT+PEDT+drug delivery	BaTiO3 ciprofloxacin	Light	BaTiO_3_-NIR	52
	PTT+drug delivery	ICG luteolin	Light	ICG-NIR	75
**Detection**	Biosensing	QD	Chemical catalysis-	Pt-H_2_O_2_	52, 53
	Bioimaging	TPE derivatives	Enzyme	Urease	33
**Capture and isolation**	Detoxication	platelet	Light magnetic	CdTe-VIS Fe_3_O_4_	15, 16
		PCL、PLAG	Magnetic	Magnetic helical nanomotor	21
	Microorganism capture	BiVO4-yeast	Light	BiVO4-VIS	99
		Lectin-E. coli Anti-protein A antibody bioreceptor-S. aureus bacteria	Ultrasound Magnetic	Au Ni	21
		ACE2 receptor-SARS-CoV-2	Bio-hybrid	Microalgae	45
		L-serine-E. coli	Chemical catalysis-	Mg-H_2_O	29

## Abbreviations

Alg: Alginate
AIE: Aggregation-induced emission
CDT: Chemodynamic therapy
Chi: Chitosan
CLR: Clarithromycin
CIP: Ciprofloxacin
E. coli: Escherichia coli
EMNMs: Enzyme-powered micro/nanomotors
GI: gastrointestinal
H_2_O_2_: Hydrogen peroxide
ICG: Indo cyanine green
JPL-motor: Janus platelet micromotor
LPS: Lipopolysaccharide
MNMs: Micro/nanomotors
MOF: metal organic framework
NRs: Nanorods
NO: Nitric oxide
NPs: Nanoparticles
NIR: Near-infrared
PABA: Para-amino benzoic acid
PB: Prussian blue
PCR: polymerase chain reaction
PCL: polymer polycaprolactone
PDA: Polydopamine
PDT: Photodynamic therapy
PLGA: Poly (lactic-co-glycolic acid)
PMB: Polymyxin B
PTAs: photothermal agents
PTT: Photothermal therapy
QDs: Quantum dots
ROS: Reactive oxygen species
TPE: Tetraphenylethene
UV: Ultraviolet
VIS: Visible
